# Gulf Coast Ticks (*Amblyomma maculatum*) and *Rickettsia parkeri*, Ohio, USA, 2020

**DOI:** 10.3201/eid3209.260632

**Published:** 2026-09

**Authors:** Risa Pesapane, Kaylee Shrock, Cece Bryant, Andrew J. Rosendale

**Affiliations:** The Ohio State University, Columbus, Ohio, USA (R. Pesapane, K. Shrock, C. Bryant); Mount St. Joseph University, Cincinnati, Ohio, USA (A.J. Rosendale)

**Keywords:** *Amblyomma maculatum*, *Rickettsia parkeri*, tick-borne diseases, ticks, host, disease vectors, Gulf Coast ticks, zoonoses, vector-borne infections, bacteria, Ohio, United States

## Abstract

We report the establishment of the Gulf Coast tick (*Amblyomma maculatum*) and *Rickettsia parkeri* bacteria in Ohio, USA. We collected host-seeking ticks through surveillance in 2020–2021. Our discovery of established populations in 2 Ohio counties highlights a medically critical vector–pathogen system with serious public health implications.

The Gulf Coast tick (*Amblyomma maculatum*) is a 3-host ixodid tick of increasing medical and veterinary note (*1*). Historically, this species inhabited only coastal regions of the southeastern United States from Florida to Virginia (*2*), but its geographic range has expanded in recent years to include portions of the Midwest and Northeast (*3*–*7*). Because the geographic distribution of this tick species is expanding, so might the distribution of its associated pathogens. Gulf Coast ticks are the vector for *Rickettsia parkeri*, a causative agent of rickettsiosis (*8*).

The state of Ohio Department of Health has received sporadic reports of Gulf Coast ticks since 1990 (Figure), but the tick was not yet documented as established (the collection of >6 ticks or >1 life stage within a county during a 12-month period) (*8*). The reports were limited, involving a total of 29 Gulf Coast tick records from 22 counties, often with only 1 tick reported in any county each year. However, unverified reports of increasing Gulf Coast tick detections in southwestern Ohio prompted our investigation into whether self-sustaining populations and their associated pathogens were present.

**Figure:**
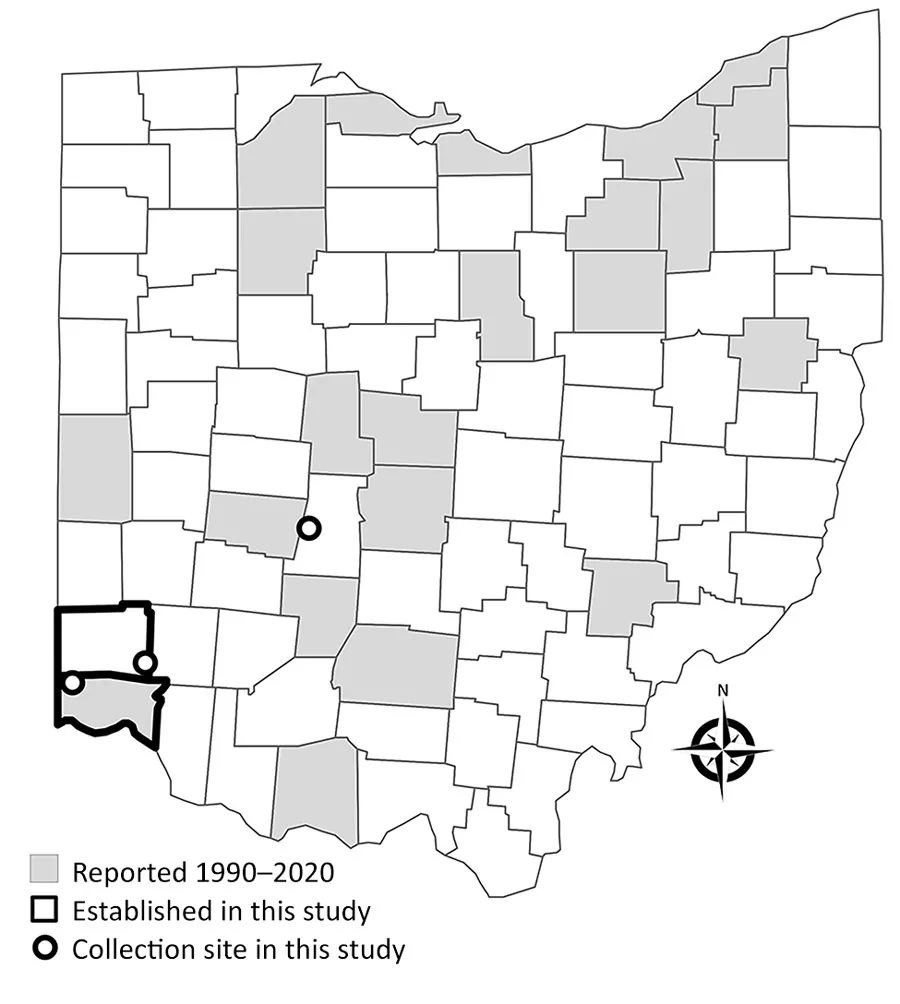
Reports of Gulf Coast ticks (*Amblyomma maculatum*) in Ohio, USA. Map highlights counties with previous reports of 1–2 Gulf Coast tick detections from the Ohio Department of Health (1990–2020), the collection sites from this study of active and passive Gulf Coast tick detections (2020–2021), and the counties we determined to have an established population (>6 adults) of Gulf Coast ticks.

We collected host-seeking ticks from vegetation in Butler County, Ohio, USA, in July 2020 and Hamilton County, Ohio, USA, in September 2020 by flagging or dragging vegetation along walking trails in public recreational areas (Appendix). We also received unfed ticks collected by a citizen scientist at a neighborhood green space in Madison County, Ohio, USA, in June and July 2021.

We obtained 41 adult *A. maculatum* ticks from 3 counties for this study (Figure): 30 (8 male, 22 female) from Hamilton County, 7 (1 male, 6 female) from Butler County, and 4 (1 male, 3 female) from Madison County. The collections met the criteria for establishment in Hamilton and Butler counties but not Madison County. We extracted DNA from 37 specimens for pathogen testing by real-time PCR (Appendix). Of the 29 specimens we tested from the Hamilton County site, 15 (51.7%) were positive for *R. parkeri* bacteria.

Established Gulf Coast tick populations have recently been documented in several states outside of the species’ historical range, including Indiana in 2020 (*3*); Illinois, Connecticut, and New York in 2021 (*4*,*5*,*7*); and New Jersey in 2022 (*6*). Those reports indicate rapid range expansion of Gulf Coast ticks in the northeastern and midwestern United States. Through active surveillance, we collected enough Gulf Coast ticks to document established populations in 2 Ohio counties in 2020; passive submissions also documented the species in a third county in 2021. Those findings suggest Gulf Coast tick establishment in Ohio occurred before 2021.

We also detected *R. parkeri* bacteria in host-seeking ticks, revealing the presence of a tick vector and its primary human pathogen in Ohio. Human and animal health providers should remain vigilant for locally acquired cases of *R. parkeri* caused rickettsiosis. The Ohio Department of Health has reported spotted fever rickettsiosis cases in Hamilton and Butler counties (*9*). Although those cases are presumed to represent Rocky Mountain spotted fever associated with *R. rickettsii* bacteria from *Dermacentor variabilis* ticks, *R. parkeri* bacteria antibodies might cross-react with antigens used in routine serologic assays for other spotted fever group *Rickettsia.* Cross-reactivity would make the causative agent and vector difficult to distinguish (*10*). Our detection of *R. parkeri*–infected Gulf Coast ticks emphasizes the need to consider alternative spotted fever group rickettsiae when interpreting human spotted fever rickettsiosis surveillance data in Ohio.

Gulf Coast tick populations might have become established before 2020 but remained undetected because of limitations in active surveillance activities. Although we collected our samples in 2020–2021, they remain relevant as historical records documenting established populations rather than as transient introductions. Our records also help define the chronology of the tick species’ spread. Our findings further underscore the value of passive tick surveillance through public submission programs in which the public submits ticks for identification as a complement to active surveillance, particularly in areas with limited surveillance capability experiencing tick range expansion.

Appendix Additional information about Gulf Coast ticks (*Amblyomma maculatum*) and *Rickettsia parkeri*, Ohio, USA, 2020.
