# Supplementary material for: Gulf Coast Ticks (Amblyomma maculatum) and Rickettsia parkeri, Ohio, USA, 2020
Source: Emerg Infect Dis. 2026 Sep;32(9):1521–2. doi: 10.3201/eid3209.260632 (PMC13555725; doi:10.3201/eid3209.260632)
Supplement: Supplementary file 1 [file 26-0632-Techapp-s1.pdf]

Article DOI: https://doi.org/10.3201/eid3209.260632

EID cannot ensure accessibility for supplementary materials supplied by authors. Readers who have difficulty accessing supplementary content should contact the authors for assistance.

Gulf Coast Ticks (*Amblyomma maculatum*) and *Rickettsia parkeri*, Ohio, USA, 2020

Appendix

Additional Methods

We conducted drags and flags in 2020 with 1m^2 muslin cloths checked for ticks every 10-m at minimum, whereas publicly submitted ticks were collected and received in 2021. We immediately stored ticks in 70% ethanol then identified stage, sex, and species by examining morphological features using light microscopy in accordance with an established key (*1*). In 2023, we dried ticks identified as *A. maculatum* of ethanol and homogenized using a bead mill (Thermo Fisher Scientific, https://www.thermofisher.com) before extracting their DNA using the PureLink Genomic DNA Kit (Invitrogen, https://www.thermofisher.com). We screened DNA for *R. parkeri* using a real-time PCR assay with previously published primers and probes (*2*). We included 5 µL of DNA in a 20 µL reaction using TaqMan Master Mix (Applied Biosystems, https://www.thermofisher.com) with an initial denaturation of 95°C for 3 minutes, then 45 cycles of 95°C for 15 seconds followed by 60°C for 1 minute. We considered postive any samples with a cycle threshold ≤40 with a characteristic curve. We included PCR-grade water and a synthetic gBlock gene fragment (Integrated DNA Technologies, https://www.idtdna.com) of *R. parkeri* (KF782321.1) as controls.

References

1. Keirans JE, Litwak TR. Pictorial key to the adults of hard ticks, family Ixodidae (Ixodida: Ixodoidea), east of the Mississippi River. J Med Entomol. 1989;26:435–48. PubMed https://doi.org/10.1093/jmedent/26.5.435

2. Jiang J, Stromdahl EY, Richards AL. Detection of *Rickettsia parkeri* and *Candidatus Rickettsia andeanae* in *Amblyomma maculatum* Gulf Coast ticks collected from humans in the United

States. Vector Borne Zoonotic Dis. 2012;12:175–82. PubMed
https://doi.org/10.1089/vbz.2011.0614
